# Engulfment and Pushing of Cylindrical Liquid Nano-Inclusion by Advancing Crystal/Melt Interface: An Atomistic Simulation Study

**DOI:** 10.3390/nano13243164

**Published:** 2023-12-18

**Authors:** Atia Perveen, Hongtao Liang, Dmitri V. Alexandrov, Muhammad Umar Dad, Yang Yang

**Affiliations:** 1 School of Physics and Electronic Science, East China Normal University, Shanghai 200062, China; 2 Research and Development Department, Zhangjiang Laboratory, Shanghai 201204, China; 3 Laboratory of Multi-Scale Mathematical Modeling, Department of Theoretical and Mathematical Physics, Ural Federal University, Lenin Ave., 51, 620000 Ekaterinburg, Russia; 4 Institute of Materials Research (iMR), Tsinghua Shenzhen International Graduate School (TSIGS), Tsinghua University, Shenzhen 518055, China

**Keywords:** molecular dynamics simulation, solidification, liquid inclusion, engulfment and pushing, Al-Pb alloy

## Abstract

We reported a molecular dynamics (MD) simulation study of an advancing pure Al(100)/melt interface that encounters a foreign immiscible liquid Pb cylindrical nano-inclusion. When the advancing interface approaches the inclusion, the interface may engulf, push to an extent and then engulf or push the nano-inclusion away from the solidifying phase depending on the velocity of the interface. Here, we investigated cylindrical liquid Pb nano-inclusion pushing or engulfment by a growing crystal Al that strongly depends on the velocity of the crystal/melt interface, and a critical velocity ($v_{c}$) is deduced. If the velocity of the interface is less than $v_{c}$, then the inclusion is pushed and engulfed otherwise. The relationship between $v_{c}$ and the radius of the nano-inclusion is expressed using a power function that agrees well with the previous studies. For velocity above the $v_{c}$, the crystal/melt interface plays a vital role; it hinders the matrix atoms from setting below the cylindrical nano-inclusion due to insufficient mass transfer below the inclusion, resulting in the engulfment.

## 1. Introduction

The solidification process is intricate, involving a multitude of factors that exert influence, one of which is the presence of foreign particles or droplets at the interface between the crystal and melt. It is imperative to delve into the intricacies of these interfaces and how they engage with foreign substances, as this comprehension holds the key to exercising precise control and achieving optimization in solidification processes across diverse materials.

It is a multifaceted phenomenon when an advancing interface approaches the foreign particles present in the molten phase; during solidification, the moving interface can push the particle, engulf it or push up to a certain distance before engulfment. The study of particle behavior at interfaces has been a topic of interest for over a century with numerous theories and studies attempting to predict this phenomenon [1,2,3,4,5,6,7,8]. The interaction between immiscible foreign particles and interfaces is encountered in numerous technological and natural situations, such as bio-inspired composites fabrications [9], the fabrication of superconductors, metal–matrix composites [1,10,11,12,13], alloy casting [1,12,14], the cryogenic preservation of tissues or cells [15,16], composites reinforced with ceramic particles [17], frost heave or ice lenses formation in frozen soils [18] and the food industry [19]. In certain applications, there is a need for solidifying fronts to actively propel particles, while in others, the engulfment of particles is crucial to achieving a uniform distribution within the matrix. For instance, consider the process of deoxidizing molten steel before solidification. In this context, the physical properties of the cast metal depend on the complete engulfment of deoxidation products. On the contrary, in specific material processing scenarios, it is essential to maintain a particle-free matrix to enhance both electrical and mechanical properties. This necessitates the active exclusion of second-phase inclusions.

The physics behind the engulfment and pushing phenomenon is extensively studied and experimentally investigated [20,21,22]. An X-ray transmission microscope is used to provide benchmark data for opaque matrix materials [23]. Experimentally observing these interactions poses several challenges, such as the potential for foreign particles to contact the cell floor, leading to additional friction. Additionally, in some cases, the similarity in the refractive index between the particle material and the matrix makes observation difficult. Molecular dynamics (MD) simulation techniques can serve as a powerful tool for microscopically studying the interaction of non-reactive foreign particles with the advancing interface.

It is widely acknowledged that the engulfment or pushing of liquid droplets [24], solid particles [25,26], or gas bubbles [5] by a solidifying front is determined by the balance of forces acting on the foreign particle, primarily the microscopic force balance. This balance reveals a maximum growth velocity, often referred to as the critical velocity ($v_{c}$), at which the particle is pushed. The behavior of particles at the interface can be transfigured into the characterization of $v_{c}$, and once $v_{c}$ is determined, engulfment or pushing can be qualitatively predicted. More broadly, $v_{c}$ is a function of the particle geometry (roughness, shape and size) [20,27,28], the thermal conductivity of the particles and the matrix material [20] as well as the surface free energy of solid–particle, crystal–melt and liquid–particle interfaces [12,25].

In the context of particle pushing, it is crucial to have a substantial force acting to prevent particle engulfment along with efficient mass transfer of the matrix liquid between the interface and the particle. This mass transfer is essential for the uniform advancement of the interface. When mass transfer does not occur rapidly enough to support the interface’s even progression, the interface’s shape can become unstable, leading to particle engulfment. It is important to note that the shape of the crystal/melt interface is highly dependent on the growth velocity, which further underscores the intricate balance between forces, mass transfer, and growth dynamics in solidification processes [28]. The shape of the crystal/melt interface is relative to the thermal conductivity of the matrix material and particles; it plays a vital role for pushing as it allows fluidic mass transportation between the crystal/melt interface and particle. Three main factors were well recognized that determine the shape of the interface, i.e., the matrix material temperature gradient, the Gibbs–Thomson effect and the dis-joining pressure. For example, the shape of the migrating crystal/melt interface is assumed to be a parabola near the particle and plainer in the region away from the particle. It has been determined by Bolling and Cissé [20] and later Chernov et al. [29,30], predicting its $v_{c}$ dependence. Gilpin [31] numerically determined the shape of the crystal/melt interface, and similar results were carried out by Aubourg [32].

Despite extensive studies on the pushing and engulfment of a solid phase foreign particle, there is a notable gap in the literature concerning the specific phenomenon of liquid nano-inclusion pushing by an advancing crystal/melt interface. The behavior of a foreign nano-inclusion in the context of an advancing crystal/melt interface remains largely unexplored. To address this research gap, we present an investigation of the cylindrical liquid nano-inclusion interactions with an advancing crystal/melt interface in the context of an Al-Pb alloy system. Our study aims to provide fundamental insights into the dynamics and mechanisms involved in nano-inclusion pushing and engulfment by the solidifying phase.

## 2. Simulation Methodology

To model Al-Pb interatomic interactions, a glue-type empirical many-body potential [33] is employed. This potential is fitted to physical quantities and experimental data; it uses a force-matching method [34] for Al-Pb cross-pair interaction and also has good agreement with the experimental phase diagram up to 1200 K. This many-body potential has successfully been applied to study Pb inclusions melting in an Al matrix [35] and for the calculation of Al-Pb interfacial free energy at low temperature [36]. It has also been thrivingly applied for the atomistic characterization of the chemically heterogeneous Al-Pb solid–liquid interface [37] and interface migration in Al [38].

We used large-scale Atomic/Molecular Massively Parallel Simulator (LAMMPS) [39] to preform all MD simulations in this work. Simulations are performed using a Nosé-Hoover thermostat at certain temperature with a thermostat relaxation time of 100 MD timesteps; the timestep is 1 fs. During simulations, a surface is created perpendicular to the interface to ensure that there is only a solid/melt interface, and the crystal will grow in only the *z* direction; periodic boundary conditions are applied across the cross-sectional (xy) directions. To regulate the pressure in the simulation box, we employed an Anderson barostat [40].

We performed non-equilibrium MD (NEMD) for directional solidification. Melting points for Al and Pb have been reported in Ref. [41]. We independently calculated the lattice constant *a* of Al at different temperatures (between 800 and 920 K) that have agreement with previous studies [37,42]. To produce the starting configuration of Al (matrix material) and Pb (foreign cylindrical nano-inclusion), we first independently generated Al atoms at a specific lattice constant that is tied up to the corresponding temperature. We simulated liquid Pb and Al separately, and each sample was run for 5 ns, and using the radial distribution function, we made sure that all the atoms are in a liquid state. A cavity is generated in molten Al, and Pb liquid inclusion (cylindrical shape) atoms of specific radius are then filled in that cavity; then, we conjoin melt (molten Al including the cylindrical liquid Pb nano-inclusion) of the same cross-section using a minimum energy separation distance with solid Al [43]. After assembling the interface, we made sure that the direction perpendicular to the Al(100)/melt interface, the *z*-axis, has enough surface or vacuum to compensate for the volume change during the solidification process, and the simulation box along the xy plane is free from constrain in the form of residual stresses within both the original and newly-grown crystal layers.

**Figure 1:**
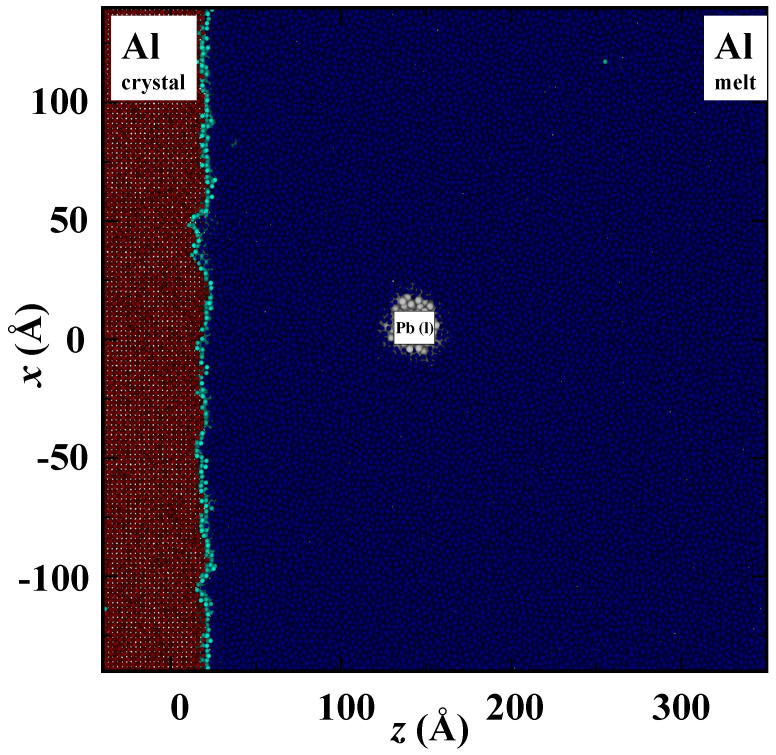
Snapshot of initial configuration of Al(100) crystal/melt interface and the Pb nano-inclusion embedded in the melt phase of Al. Al atoms are colored using order parameters (ξ). Al atoms with ξ close to 1 are in the solid phase and colored red, Al atoms with ξ close to 0 are in the liquid phase and colored blue. The interfacial Al atoms have ξ near to 0.5 and are colored cyan. Liquid Pb nano-inclusion is colored white.

We simulated five different radii ($r_{p}$) of the cylindrical liquid Pb nano-inclusion 12 Å, 16 Å, 20 Å, 30 Åand 36 Å in the Al matrix at different temperatures. The initial simulation box setup is shown in Figure 1 and more details about the simulation box are listed in Table 1. There are computational advantages for considering a semi-2D cylindrical droplet in the atomistic simulations [44] compared with the 3D spherical droplet. The symmetry along the cylinder axis (*y*) allows periodic boundary conditions to be applied in the *y* direction. The simulation box dimensions are approximately 300 Å × 32 Å × 400 Å. The simulation cell in the *y* direction is chosen to be wide enough such that there are no observable size effects on the values of different measurements. We found that $L_{y}>30$ Å gives results within the uncertainty of these measurements. A larger inclusion radius $r_{p}$ can be simulated with fewer atoms, since the inclusion volume scales as $r_{p}^{2}$ in the cylindrical geometry instead of $r_{p}^{3}$ for the spherical geometry. With larger inclusion sizes, this enables us to examine more pushing–engulfment critical transitions by including inclusion $r_{p}$ over 3 nm in pushing simulations. As described previously [45,46,47,48,49,50], it allows a larger inclusion radius to be studied using the same number of particles than in the spherical geometry, and the main physics obtained from the simulation, i.e., the interfacial thermodynamics, transport, and dynamical properties, could be successfully served for the validation of the existing predictive theories and the interpretation of the experimental observations.

During NEMD runs, we started with NVT simulations having surface along the *z*-axis, and to characterize the dynamic, thermodynamic and structural properties along the interfacial normal direction, we determined a number of interfacial profiles that can help us to analytically observe engulfment and the pushing of cylindrical liquid Pb nano-inclusion.

For the fine-scale density profiles we determined, $\rho_{i}\left(z\right)$, i= Al or Pb, is determined by averaging the number of atoms in each bin of spacing Δz divided by the volume of discrete bin, $V_{z}$,

(1) $$\rho_{k}\left(z\right)=\frac{\langle N_{z}^{k}\rangle }{V_{z}},$$

where $V_{z}=A_{xy}\Delta z=L_{x}L_{y}$ and $\langle N_{z}^{k}\rangle$ is the average number of atoms in a bin. The bin size Δz for the simulation box is chosen as 0.01Å for all simulations.

In order to observe the phenomenon of engulfment and pushing, we first need to determine the position of the advancing crystal/melt interface. For this purpose, we determined the local structural order parameters for all atoms. The state of the solid is different than that of the liquid; we used the order parameter determined by Morris et al. [51] that distinguishes crystal and melt atoms. Order parameters are determined by characterizing the local environment of an atom, in a perfect fcc lattice for any vector r connecting near neighbors, the set of $N_{q}$ wave vectors $q_{i}$ is chosen as

(2) exp(iq·r)=1

We omit the set of anti-parallel vectors for the fcc lattice; thus, $N_{q}=6$. Then, the local order parameter is defined as

(3) $$\xi ={\left|\frac{1}{N_{q}}\frac{1}{Z}\sum_{r}\sum_{q}\exp\left(iq\cdot r\right)\right|}^{2},$$

in which the sum on vector r runs over all the neighboring atoms *Z* that are within a cutoff distance $r_{c}$, while $r_{c}$ is the midpoint between the first and second-neighbor shell in a perfect fcc lattice. The order parameter is equal to one for a perfect fcc and has close-to-zero values for atoms in the melt phase. At finite temperature, solid atoms possess significant atomic vibrations that cause a reduction in order parameters, so we calculated an average order parameter for better distinguishing solid-phase atoms from liquid-phase atoms,

(4) $${\bar{\xi }}_{i}=\frac{1}{Z+1}\left(\xi_{i}+\sum_{j}\xi_{j}\right),$$

here, *j* runs over all neighbors of *Z* of atom *i* within $r_{c}$.

After determining the order parameters for each atom, the crystal/melt interface position can be defined via fitting the order parameter profile to a hyperbolic tangent function,

(5) $$f\left(z\right)=c_{1}+c_{2}\tanh\left(\frac{z-c_{3}}{c_{4}}\right),$$

where $c_{i}$ represents the fitting parameters. We will use the crystal/melt interface position $c_{3}$ to determine the crystal/melt interface migration velocity. When the interface approaches the foreign particle, the crystal/melt interface migration velocity is limited by the foreign particle being pushed, and it will be same as the pushing velocity of a foreign particle. The position of the center of mass of liquid Pb nano-inclusion is determined and is further used to calculate the pushing velocity or the crystal/melt interface migration velocity.

To improve the statistics of our MD analysis, we performed 40 replica simulations with the same initial configuration but different initial velocities. Each engulfment and pushing, simulation starts from the initial configuration with randomly distributed velocities of Pb nano-inclusion and liquid Al at the desired temperature. Atomic trajectories are sampled after every 1000 MD steps for the examination of the interface shape, the crystal/melt interface position, the pushing velocity, etc.

## 3. Results

In this research, we conducted a comprehensive molecular dynamics (MD) simulation study focused on the interaction between a pure Al(100)/melt interface and an immiscible Pb nano-inclusion. Our investigation primarily centered around the behavior of this nano-inclusion when confronted by the advancing solidification interface. We observed that the interaction dynamics were critically influenced by the velocity of the interface.

**Figure 2:**
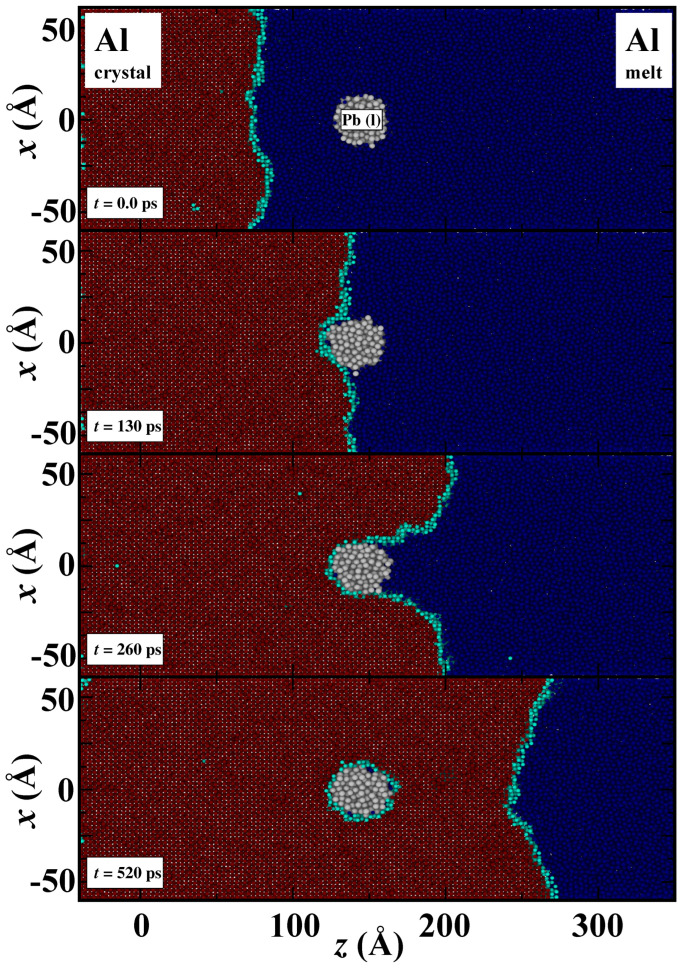
Snapshots of engulfment of liquid Pb by the advancing Al(100)/melt interface at 800 K. The color coding for atoms is the same as in Figure 1. In the top panel, a rough and plane interface is advancing toward the nano-inclusion, in the second top panel, it approaches the nano-inclusion, and in the third panel, after 26 ps, the interface become curved. In the bottom panel, nano-inclusion is engulfed by the solidification interface.

**Figure 3:**
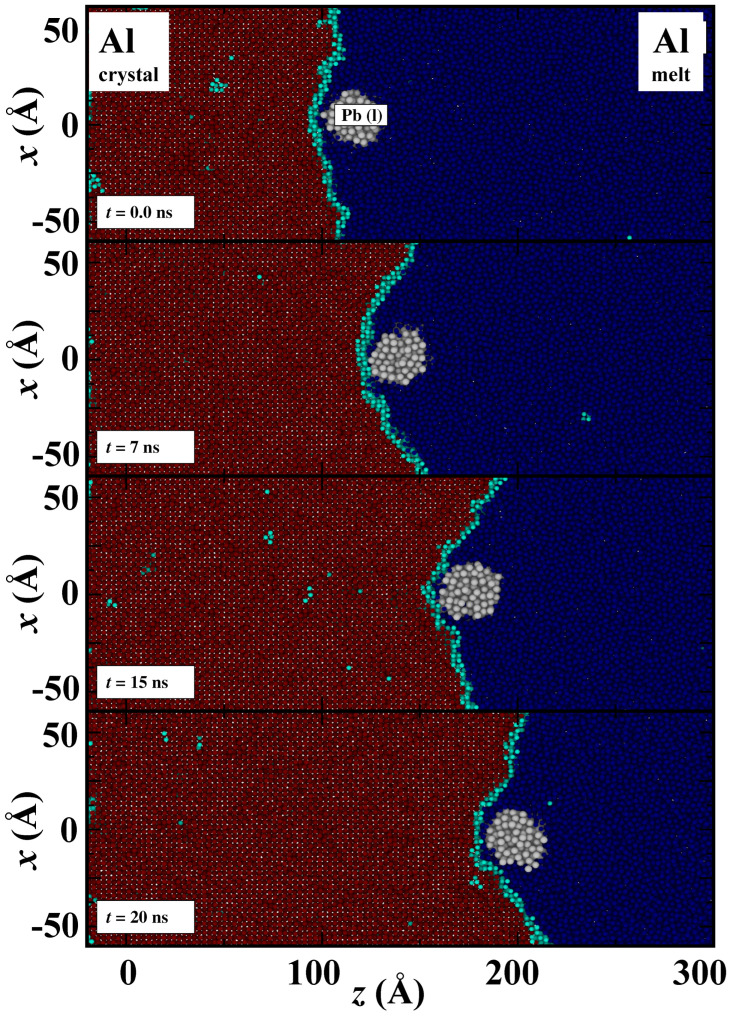
Snapshots for pushing of liquid Pb nano-inclusion by the advancing Al(100)/melt interface at 920 K. The color coding for atoms is the same as in Figure 1. The interface is advancing along the *z*-axis, and four snapshots at 0 ns, 7 ns, 15 ns and 20 ns are shown, respectively, from top to bottom. Nano-inclusion is pushed 9 nm in distance along the *z*-axis within a time window of 20 ns.

When the advancing interface neared the nano-inclusion, we observed two distinct behaviors: engulfment and pushing. The specific outcome, whether the inclusion was engulfed, pushed away, or a combination of both, depended significantly on the velocity of the crystal/melt interface. The simulation results of engulfment and pushing are shown in Figure 2 and Figure 3, respectively. Here, crystalline Al atoms are represented by red color, liquid Al atoms are represented by blue, inter-facial Al atoms are represented by cyan and liquid Pb atoms are represented by white. The colors of Al atoms depend on the order parameters. For instance, if an atom is in a liquid state, its local order parameter value will be close to zero, and we assign it a blue color. At an interface where atoms are converting from melt to crystal and the order parameter value is around 0.5, these atoms are colored cyan. Finally, when Al atoms solidify and the order parameter is one, then Al atoms turned red. In Figure 2, the nano-inclusion is engulfed, and the simulation box temperature is set to 800 K (greater undercooling and thermodynamic driving force for solidification). In Figure 3, the nano-inclusion is not engulfed throughout the simulation, and the temperature is 920 K (small undercooling and thermodynamic driving force for solidification). The semi-2D structure of xz planes offers a clear view regarding the directional solidification as well as the interaction between the advancing crystal/melt interface and the foreign nano-inclusion.

**Figure 4:**
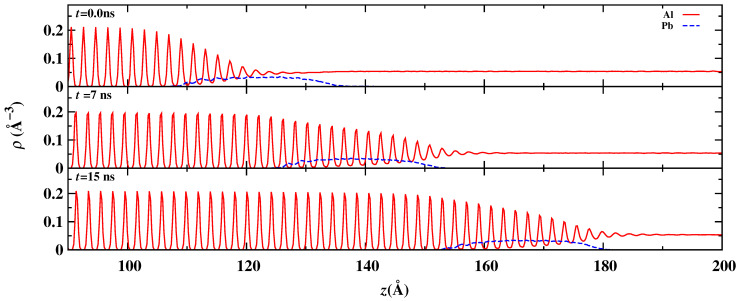
The instantaneous fine-scaled density profiles of the Al and Pb species during the NEMD. Top panel, density profiles are captured when the Pb nano-inclusion is started and being pushed at a steady-state rate. At this position, the simulation time is considered as 0.0 ns. Middle panel, density profiles are extracted based on the simulation trajectory after 7 ns. Bottom panel, density profiles are extracted based on the simulation trajectory after 15 ns.

The fine-scale density profile for the advancing Al(100) crystal/melt interface with liquid Pb nano-inclusion of the 16 Å radius is shown in Figure 4. The solid line indicates the Al density in a specific plane, and the dashed line represents the Pb density. The crystalline Al has density peaks corresponding to the lattice planes. The heights of these density peaks decrease as the crystal/melt interface is approached, and the density oscillation becomes negligible in the molten region. At the crystal/melt interface, tiny peaks overlap the density of Pb because of the curved interface. As we can see in Figure 3, that interface is not planar (but curved) near the Pb nano-inclusion.

Initially, we run 40 replica simulations for each radius with different temperatures from 800 to 920 K (melting point of Al is 922.42 K for the EAM potential used for this study). In all of our simulations, the matrix material is Al and the miscibility with Pb is negligible, and there is no solute drag effect [52]. For the lower temperatures, nano-inclusion instantly engulfed and from 906 to 909 K, there is a transition from engulfment to pushing of the nano-inclusion. We observed that Al crystal grew at high velocity in the beginning of the simulation in which the crystal/melt interface is far from the nano-inclusion. When the crystal–melt interface approached the nano-inclusion, it slowed down due to the short-range structural forces between the nearby crystal–melt interface and the heterogeneous liquid–liquid interface [42].

**Figure 5:**
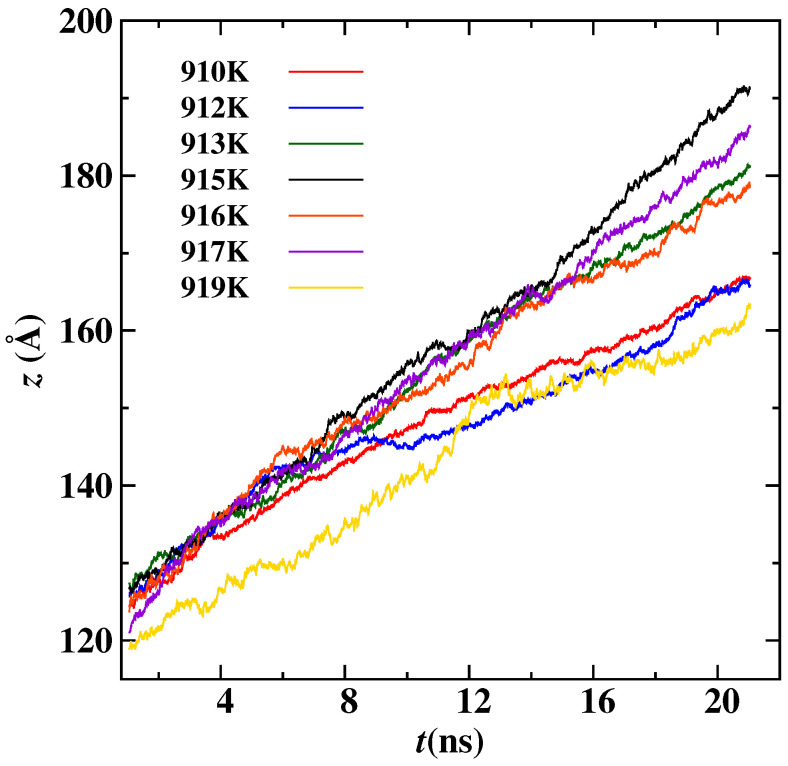
The positions of center of mass of 16 Å Pb nano-inclusion as the function of MD simulation time. Seven different simulation cases at different temperatures are plotted.

**Figure 6:**
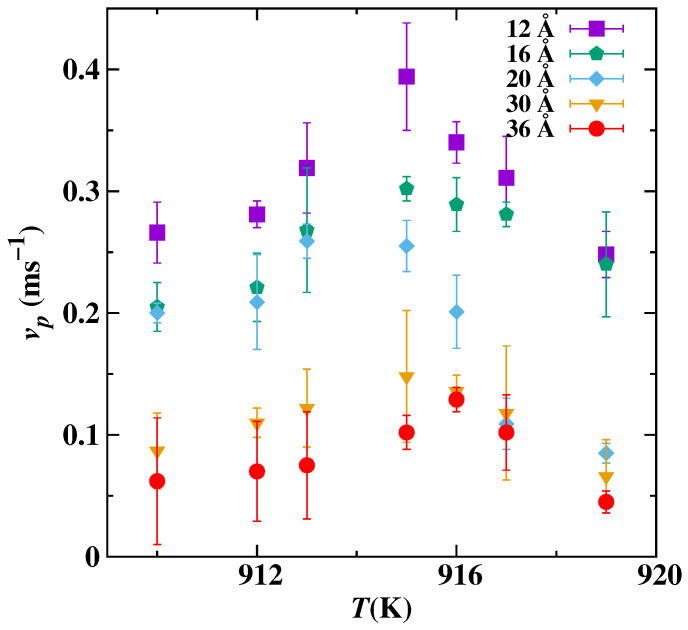
The pushing velocity $v_{p}$ of the Pb nano-inclusion (five different inclusion radiuses) as functions of simulation temperature.

As the interface started pushing the immiscible nano-inclusion, then the real-time position is tracked using the center of mass of Pb, as shown in Figure 5. A 16 Å Pb nano-inclusion is pushed ahead of the interface at seven different temperatures; see Figure 5. To calculate the pushing velocity $v_{p}$ of nano-inclusion, we determined the gradient of distance covered by the center of mass with respect to time. This pushing velocity is the same as the crystal/melt interface velocity. In all of the five radius cases, the nano-inclusions were being pushed if the temperature was above 910 K, and the $v_{p}$ is shown in Figure 6. In Figure 6, when the temperature is high and near the melting point, then $v_{p}$ is low. When cooling increases, $v_{p}$ also increases up to an extent: for instance, $v_{p}$ for the 12 Å radius is low at 919 K, and its value increases as the temperature decreases to 915 K. This is because of the increase in driving force with undercooling. But with a further decrease in temperature from 915 K, the $v_{p}$ also decreases; this is because the interface become more curved and it takes more time for Al atoms to diffuse under the nano-inclusion. This trend of increasing in $v_{p}$ with undercooling up to an extent and decreasing is similar for the remaining magnitude of the radius cases. For the same temperature, smaller nano-inclusion radius cases have overall higher $v_{p}\left(T\right)$ relationships.

In Figure 6, for certain nano-inclusion radiuses, there is the highest pushing velocity near T=915 K in each $v_{p}\left(T\right)$ dependency. Here, we refer to this as critical velocity ($v_{c}$). A number of theoretical models have been introduced to predict the interaction of foreign particle with interface as the function of both particle radius and the temperature, i.e., $v_{p}\left(T,r_{p}\right)$ dependencies [53]. These models provide a quantitative prediction of $v_{c}$ for the foreign particle at the advancing interface. Above a certain critical velocity $v_{c}$, the particle will be engulfed as shown in Figure 2, and below that, the particle will be pushed as in Figure 3. For example, Shangguan et al. [17], Stefanescu et al. [54], Kim and Rohatgi [55], and Azouni et al. [56] predict that critical velocity is reciprocal to the radius of nano-inclusion. On the other hand, Uhlmann et al. [28], Chernov et al. [29], and Cisse et al. [20] proposed that the velocity is reciprocal to $r_{p}^{2}$, $r_{p}^{3/4}$ and $r_{p}^{3/2}$, respectively. Considering the exponents among different models vary, we could summarize a $v_{c}\left(r_{p}\right)$ relationship using a power function with a variable exponent:

(6) $$v_{c}=Mr_{p}^{-n},$$

in which *M* is a pre-factor constant and *n* is the exponent, which have different values for different models. Some reported values of *M* and *n* are given in Table 2.

**Figure 7:**
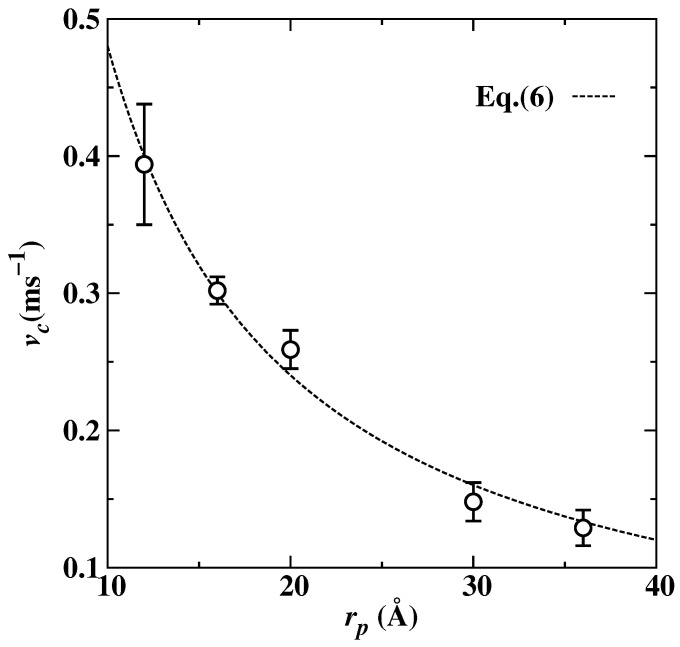
The determined critical velocity $v_{c}$, the pushing–engulfment transition velocity, as a function of radius of nano-inclusion. The dashed line corresponds to the analytic model as expressed in Equation (6).

We analytically determined the $v_{c}$ from the $v_{p}\left(T\right)$ data for different nano-inclusion radius cases, thus obtaining the relationship between the $v_{c}$ and $r_{p}$ (radius of the nano-inclusion). In Figure 7, critical velocity $v_{c}$ decreases with the increase in $r_{p}$. The dashed line in Figure 7 is the power function described in Equation (6). Through weighted least-square fitting, the estimated values of *n*, and *M* (see in Table 2) have good agreement with previous studies.

**Figure 8:**
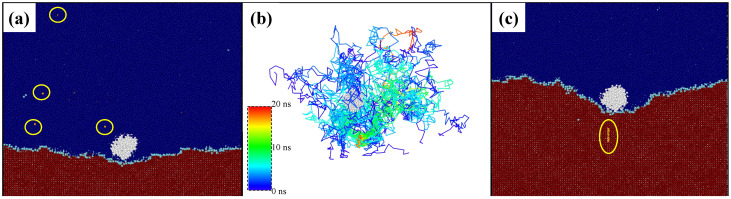
(**a**) MD snapshot of advancing Al(100) crystal/melt interface that encounters the Pb nano-inclusion. Color-coding scheme is as shown in Figure 1. Yellow-colored highlighted atoms are molten Al atoms that are distributed randomly. (**b**) The trajectories of these chosen Al atoms as the function of simulation time are plotted. (**c**) After 20 ns, these atoms solidified in the newly grown crystal behind the nano-inclusion, which is pushed ahead by the interface.

Next, we employ an analysis to reveal microscopic kinetics of interface motion during the engulfment and pushing. We track atoms in time and space, as shown in Figure 8. We identified 20 atoms that will finally diffuse behind the Pb nano-inclusion and assigned them an orange color. In Figure 8a, some Al atoms are circled for reference, and then we tracked the path of these atoms along with other identified atoms. We noted the Al(100) crystal/melt interface is rough during solidification. Each site along the rough crystal/melt interface is a potential site for the attachment of atoms during solidification; the rate of attachment of atoms to the interface corresponds to the Brownian motion of the atoms in the molten phase. Simply, the time required for an atom to solidify is proportional to the minimum time expected from its random walk. In Figure 8b, we identified the path of 20 atoms, and their trajectory is colored with respect to time scale. In the beginning of the simulation, the trajectory is colored as dark blue; it changes to green after 10 ns; and when the atoms totally become crystal under the Pb nano-inclusion, it is colored red.

We did not find any diffusion barrier during solidification. Since the atom moves freely in the melt by Brownian motion, the displacement of the boundary corresponds to a biased random walk. Here, bias is a result of the difference in temperature of crystal and melt. The driving force for this based random walk can be characterized by Brownian velocity for instance at any time τ an atom of mass *m* has Brownian velocity $v_{\mathrm{rw}}=\lambda /\tau$; here, λ is the jump distance. An equipartition principle application to kinetic energy, $k_{B}T/2=mv_{\mathrm{rw}}^{2}/2$, gives $m=k_{B}T/v_{\mathrm{rw}}^{2}=k_{B}T\tau^{2}/\lambda^{2}$ [60]. The transformation of atoms from melt to crystal or set under the Pb nano-inclusion are driven by the difference of chemical potential, ΔG, that is due to the concentration gradient. The gradient of chemical potential gives driving force, $F_{x}=\nabla G=\Delta G/\lambda$. One can determine the drift velocity of the interface using Newton’s third law $v_{D}=\frac{F_{x}\tau }{2m}=\frac{\Delta G\lambda }{2k_{B}T\tau }$ [60] in response to the driving force. Going into more detail of Brownian motion of atoms is beyond the scope of this study, but we believe it is worth studying because such a drift velocity of the interface $v_{D}$ can be controlled by *T*, and if there is not enough motion of atoms to set behind the Pb nano-inclusion, as shown in Figure 8c, then pushing of the Pb nano-inclusion is very difficult.

Not only does the Brownian motion of melt atoms impact the migration of the foreign nano-inclusion; other factors like the interface shape and the distance between the advancing crystal/melt interface and the nano-inclusion also play a role. A number of theoretical analyses have been conducted to develop equations describing the shape of the interface and the temperature field in the vicinity of foreign particles where the interface is assumed to follow the isotherm of the matrix material for melting. Experientially, techniques like synchrotron X-ray radiography, the state-of-art electron microscope and energy-dispersive X-ray spectroscopy can capture in real time the interfacial evaluation in metallic alloys. But these techniques have not been yet applied to immiscible alloys containing liquid droplets of nano size because of additional challenges that arise from Marangoni flows, droplet coalescence, diffraction-limited spatial resolution as well as the Brownian motion. According to the best of our knowledge, to date, there are no direct simulations or experimental studies that capture the phenomenon of engulfment and pushing in immiscible metallic alloys that contain the liquid nano-inclusions. Despite the lack of direct observation on the immiscible liquid nano-inclusion at the advancing interface, our simulation results provide detailed microscopic insight, which could benefit the improvement of the related thermodynamic and kinetic theories of the solidification.

## 4. Summary

In summary, we have presented a simulation study of the intriguing phenomenon of liquid lead nano-inclusion engulfment and pushing by an advancing crystal/melt interface of pure aluminum. The fate of the Pb nano-inclusion was found to be intricately linked to the growth velocity of the crystal/melt interface. In cases where the interface advanced at low undercooling conditions, the nano-inclusion was observed to be continuously pushed along the solidification direction. As the interface velocities surpassed a critical threshold, a transition occurred; then, the nano-inclusion was engulfed by the advancing crystal/melt interface, resulting in a liquid inclusion embedded in the Al matrix.

One key contribution of this study was the determination of the critical velocity, which serves as a pivotal parameter governing the engulfment and pushing behavior. We validated that the critical velocity of the liquid metal nano-inclusion (pushed by the crystal/melt interface) followed a power law relationship with the radius of the nano-inclusion, which is in agreement with previous theoretical models made for predicting pushing–engulfment transition phenomena.

The current study provides valuable insights into the intricate mechanisms underlying the engulfment and pushing phenomena. Our findings provide valuable insights into crystal growth processes and open up new avenues for tailoring material properties through controlled particle interactions at the interface. The knowledge gained from this study holds promise for numerous practical applications. The inverse relationship between the critical velocity and nano-inclusion radius can be utilized to inform the addition of de-oxidizers in metal matrices, enabling more effective control of the properties of materials. Conversely, in material processing scenarios where it is desirable to maintain a matrix free from second-phase inclusions to enhance electrical and mechanical properties, understanding the dynamics of particle pushing becomes crucial.

## Figures and Tables

**Table 1:** Summary of the simulation details, including the radius of the Pb nano-inclusion, and the number of Al and Pb atoms.

$r_{p}$ Å	$N_{\mathrm{Al}}$	$N_{\mathrm{Pb}}$
12	158,100	445
16	157,704	687
20	156,778	1218
30	154,135	2730
36	151,629	5918

**Table 2:** Summary of different matrix/particle systems, a comparison of the pre-factor *M* and exponent *n* used in Equation (6) for describing the particle radius dependent of the critical velocity for a engulfment–pushing transition.

Matrix/Particle System	*n*	$M,\mu m^{2}/s$
Biphenyl/acetal	0.90	1132 [27]
Biphenyl/nylon	0.64	199 [27]
Naphthalene/acetal	0.30	195 [27]
Naphthalene/nylon	0.46	142 [27]
Succinonitrile/polystyrene	1.0	12.1 [57]
Steel/silica–alumina (liquid)	1.0	24 [58]
Aluminum/zirconia	1.0	250 [59]
Aluminum/lead (liquid)	1.0	347.6

## Data Availability

The data that support the findings of this study are available from the corresponding author, M.U.D. or Y.Y., upon reasonable request.
